# Novel Insights into the Links between N6-Methyladenosine and Regulated Cell Death in Musculoskeletal Diseases

**DOI:** 10.3390/biom14050514

**Published:** 2024-04-24

**Authors:** Juanjuan Han, Cuijing Wang, Haolin Yang, Jiayi Luo, Xiaoyi Zhang, Xin-An Zhang

**Affiliations:** 1 College of Exercise and Health, Shenyang Sport University, Shenyang 110100, China; 2111517001@sus.edu.cn (J.H.); wangcuijing2024@163.com (C.W.);; 2 College of Pharmacy, Jilin University, Changchun 132000, China; 13089243676@163.com; 3 College of Second Clinical Medical, China Medical University, Shenyang 110100, China; zhang15840054840@163.com

**Keywords:** regulated cell death, musculoskeletal diseases, m6A, apoptosis, autophagy-dependent cell death, ferroptosis, pyroptosis

## Abstract

Musculoskeletal diseases (MSDs), including osteoarthritis (OA), osteosarcoma (OS), multiple myeloma (MM), intervertebral disc degeneration (IDD), osteoporosis (OP), and rheumatoid arthritis (RA), present noteworthy obstacles associated with pain, disability, and impaired quality of life on a global scale. In recent years, it has become increasingly apparent that N6-methyladenosine (m6A) is a key regulator in the expression of genes in a multitude of biological processes. m6A is composed of 0.1–0.4% adenylate residues, especially at the beginning of 3′-UTR near the translation stop codon. The m6A regulator can be classified into three types, namely the “writer”, “reader”, and “eraser”. Studies have shown that the epigenetic modulation of m6A influences mRNA processing, nuclear export, translation, and splicing. Regulated cell death (RCD) is the autonomous and orderly death of cells under genetic control to maintain the stability of the internal environment. Moreover, distorted RCDs are widely used to influence the course of various diseases and receiving increasing attention from researchers. In the past few years, increasing evidence has indicated that m6A can regulate gene expression and thus influence different RCD processes, which has a central role in the etiology and evolution of MSDs. The RCDs currently confirmed to be associated with m6A are autophagy-dependent cell death, apoptosis, necroptosis, pyroptosis, ferroptosis, immunogenic cell death, NETotic cell death and oxeiptosis. The m6A–RCD axis can regulate the inflammatory response in chondrocytes and the invasive and migratory of MM cells to bone remodeling capacity, thereby influencing the development of MSDs. This review gives a complete overview of the regulatory functions on the m6A–RCD axis across muscle, bone, and cartilage. In addition, we also discuss recent advances in the control of RCD by m6A-targeted factors and explore the clinical application prospects of therapies targeting the m6A–RCD in MSD prevention and treatment. These may provide new ideas and directions for understanding the pathophysiological mechanism of MSDs and the clinical prevention and treatment of these diseases.

## 1. Introduction

N6-methyladenosine (m6A) RNA modification is both dynamic and reactive and is controlled by three enzymes—namely, m6A methyltransferases (“writers”), m6A demethylases (“erasers”), and m6A binding proteins (“readers”)—that create an elaborate interplay between m6A incorporation, degradation, and recognition [1]. Numerous investigations have reported that m6A can influence many aspects of RNA metabolism. m6A readers can dually regulate the processing and degradation of pri-miRNAs [2]. m6A methylation modulates circRNA cytoplasmic export, stability, and biogenesis in diverse disease patterns [3]. Among mRNAs and ncRNAs, m6A is one of the most sufficient post-transcriptional modifications, encompassing the fate of the RNA in multiple phases, such as transport from the nucleus to the cytoplasm, RNA splicing, translational efficiency, and RNA stabilization [4]. Currently known regulated cell death (RCD) types mainly include autophagy-dependent cell death, apoptosis, necroptosis, pyroptosis, ferroptosis, parthanatos, entotic cell death, NETotic cell death, lysosome-dependent cell death, and oxeiptosis. They serve an essential role in the stability of homeostasis in vivo, the development of multiple systems, and the evolution of organisms [5]. RCD can occur in the absence of any exogenous environmental disturbances and can therefore act as an in-built effector of physiological procedures of development or tissue turnover. These fully physiological RCDs are often referred to as programmed cell death (PCD). Moreover, abnormal RCD is widely used to influence the evolution of several diseases, including cancer, and is receiving more and more interest [6]. Research has demonstrated that diverse categories of cell death share a coordinated system and are not independent of each other [7,8]. When there is an abnormality in one pathway, other regulatory mechanisms will ensure that cell death proceeds. Such processes have a major impact on cancer development and progression [6], such as in lung adenocarcinoma [9], breast cancer [10], thyroid cancer [11], and gastric cancer [12]. Furthermore, the m6A–RCD axis likely exerts a key role in drug therapeutic efficacy and resistance, including immune efficacy, chemotherapeutic resistance, and drug side effects. Importantly, recent evidence suggests that RCD is also regulated by m6A in musculoskeletal disorders (MSDs), playing a crucial role in the pathophysiological mechanism of MSD [13,14,15,16].

MSDs are a group of inflammatory and degenerative diseases caused by injury or pain in the locomotor organs. MSDs primarily affect the musculoskeletal system, such as muscles, bones, cartilage, and joints, and they include osteoarthritis (OA), osteosarcoma (OS), multiple myeloma (MM), intervertebral disc degeneration (IDD), and osteoporosis (OP). Bone loss and sarcopenia are the main clinical manifestations of MSDs. Currently, the exact pathogenesis of MSD remains unclear, and there are no effective treatments or therapeutic agents for it [13]. Some MSDs have effective pharmacological treatments that can reduce disease activity and thus improve disability, e.g., rheumatoid arthritis [17]. However, other MSDs have no effective therapeutic options, e.g., osteoarthritis [18]. In addition to this, lifestyle behavioral changes can help improve the progression of MSD [19]. There is growing evidence to suggest that m6A modifications are closely associated with the musculoskeletal system and are considered to be key regulators associated with the incidence and evolution of the disease. m6A-related proteins with RCD are vitally important during the pathological and physiological procedures of MSD. Furthermore, the review describes some of the roles of m6A–RCD in the tissues (muscle, bone, cartilage) of different musculoskeletal systems. Numerous studies have confirmed that the m6A–RCD axis is involved in muscle senescence [14] and energy metabolism [15], and this axis is also associated with bone metabolism [16]. In addition to this, the m6A–RCD axis affects chondrocyte viability [20,21] and migration [22]. A thorough review of the literature was carried out across multiple scholarly databases including PubMed, Web of Science, Google Scholar, and Scopus. The search strategy utilized specific terms such as “m6A”, “ferroptosis”, “autophagy-dependent cell death”, “apoptosis”, and “pyroptosis” in conjunction with “osteosarcoma”, “osteoarthritis”, and “multiple myeloma” to gather the latest research findings. We focused on critical roles that m6A regulators interacting with RCD play in the onset and progression in MSDs, while further describing the relevant molecular mechanisms of m6A and RCD links in MSDs and their possible clinical applications. We found that m6A-mediated RCD could offer a novel potentially effective target for the management of MSD.

## 2. m6A Modification

**Figure 1:**
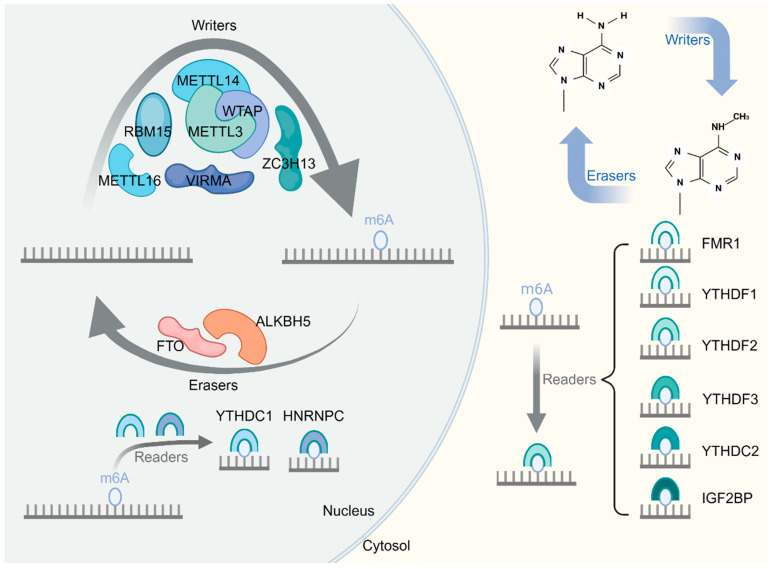
Function of m6A. m6A methylation is catalyzed by the writer’s complex, including METTL3, METTL14, WTAP, VIRMA, RBM15, and ZC3H13. In addition, the m6A modification is removed by demethylation enzymes such as FTO or ALKBH5. Furthermore, the readers, including FMR1, YTHDF1/2/3, YTHDC2, and IGF2BP, recognize m6A and determine the fate of the target RNA.

The m6A modification is a prominent posttranscriptional regulatory mechanism present in eukaryotic mRNAs and non-coding RNAs. It provides key contributions to a range of biological processes [23], including the modulation of cell differentiation, tissue development, and stress responses [24]. In addition, it has been implicated in the regulation of immune responses [25]. The course of m6A modification is controlled by three different groups of proteins, namely “writers”, “erasers”, and “readers” [26]. The “writers” include METTL3, WTAP, METTL14, KIAA1429, ZC3H13, and RBM15/15B, which are responsible for adding m6A to RNA. METTL3, also known as methyltransferase 3, is the main catalytic enzyme for m6A methylation. m6A is also formed by METTL14, or methyltransferase 14, which acts as a partner of METTL3 to form a complex in which the two work together to participate in the formation of m6A [27,28]. Although METTL14 lacks catalytic activity, it takes on a vital role in the formation on m6A by assisting in the localization of METTL3, allowing precise methylation at the correct location [29]. WTAP, an integral part of the m6A methyltransferase, acts synergistically with METTL3 and METTL14 to direct regioselective methylation [30]. KIAA1429, also known as VIRMA, is an essential component of the m6A methyltransferase complex that mediates the preferential methylation of mRNAs in area of the 3′UTR and the stop codon. VIRMA achieves this by targeting regioselective methylation through the recruitment of the catalytic core components METTL3/METTL14/WTAP [31]. RBM15 and its cognate protein, RBM15B, are constituents of the m6A methyltransferase complex and influence m6A deposition by interacting with WTAP and helping the m6A methyltransferase complex to target specific RNA locations [32]. ZC3H13, a ribonucleic-acid-binding protein, acts as a crucial player during the methylation of m6A. ZC3H13 forms a complex with other members of the m6A writing complex, such as WTAP, VIRMA, and RBM15/15B, which facilitates the formation of the m6A near the 3′UTR and mRNA stop codon. The formation of this complex contributes to the location of m6A and may affect mRNA stability and transcription efficiency [28]. Instead, ‘erasers’, like ALKBH5 and a protein known as FTO, or fat mass and obesity associated protein, function as erasers of m6A, regulating the levels of this compound through demethylase activity. FTO binds to m6A through its catalytic structural domain, using α-KG as a cofactor to remove its methyl group on m6A by redox reaction to produce n6-formyladenosine (f6A) and n6-hydroxymethyladenosine (hm6A). FTO is mainly located in the nucleus, enriched at the 3′ untranslated regions (3′UTRs) of mRNAs as well as in the vicinity of stop codons. Therefore, the activity of FTO can affect the translation efficiency and stability of mRNA, which ultimately affects gene expression. Notably, another m6A eraser, ALKBH5, operates in a similar manner to FTO [33,34]. “Readers” include YTHDC1, YTHDC2, YTHDF1, YTHDF2, YTHDF3, HNRNPC, IGF2BP1/2/3, FMR1, and LRPPRC, which have the ability to recognize m6A modifications and mediate their biological effects. YTHDF1 recognizes m6A-modified mRNAs and promotes their translation. This is achieved through its link with the translator initiation element *eIF3*, which enhances the efficient transcription of m6A-modified mRNAs. YTHDF2 contributes the degradation of m6A-modified mRNAs by recognizing them and locating them to degraded cytoplasmic processing bodies. YTHDF3 synergizes with YTHDF1 and YTHDF2 by enhancing the translation of m6A-modified mRNAs and promoting their degradation [35,36]. YTHDC1 acts in the nucleus and functions to influence the splicing of m6A-modified mRNAs. This protein interacts with *SRSF3*, a splicing factor, and prevents another splicing factor, *SRSF10,* from binding, thus affecting the splicing pattern of m6A-modified mRNAs. Similarly, YTHDC2 also affects the translation and stability of m6A-modified mRNAs through interactions with the translation initiation factor *eIF3* and RNA deconjugating enzyme DDX3, which enhances m6A-modified mRNA translation and also affects their stability [37,38]. IGF2BP1/2/3 are RNA-binding proteins that recognize and bind to m6A-modified mRNAs, thereby significantly affecting mRNA stability and translation. These proteins bind to m6A-modified mRNAs through their specific RNA-binding domains and perform essential functions in regulating gene expression by either stabilizing mRNAs or enhancing mRNA translation [39]. HNRNPC recognizes m6A-modified RNA and regulates selective splicing. The protein is known for its affinity for uridine-rich sequences in RNA. The presence of m6A induces the binding of HNRNPC, which in turn affects the selective splicing of bound RNA. HNRNPC also plays a vital part in exporting mature mRNA from the nucleus to the cytoplasm [40,41]. FMRP/FMR1 is an RNA-binding protein related to fragile X syndrome and plays an influential role in regulating synaptic plasticity. The protein is known to regulate the translation of these RNA. FMR1 can positively and negatively affect the translation of m6A-modified RNA [42,43]. LRPPRC is an m6A reader involved in the stabilization of mitochondrial mRNA by forming a complex with another RNA-binding protein, SLIRP [44,45]. Methylation modifications, termed m6A, have been detected in non-coding RNA molecules, such as micro-RNA (miRNAs) and long-stranded non-coding RNA (lncRNAs). The presence of m6A modifications in these non-coding RNA affects their stability, transcription, processing, and function. For example, m6A modifications can be observed to have an effect on the stability and cellular localization of lncRNAs, thereby affecting their involvement in the regulation of gene expression [46]. Similarly, m6A modifications can affect the biogenesis of miRNAs, which in turn affects the levels and functions of these molecules. Interestingly, it has also been found that circular RNA can be recognized and bound by m6A-reading proteins, ultimately affecting the function of circular RNA [47]. The effects of m6A modifications in different types of cancers, namely leukemia and breast and liver cancers, have been extensively studied. It has been found that the regulation of m6A can control cancer progression by affecting cell proliferation, apoptosis, and metastasis [48]. And m6A modifications have been related to many other diseases, such as cardiovascular disease, neurological disorders, and obesity [49]. In addition, the modification of m6A serves a vital aspect in the onset and evolution of various MSDs [50], including OA, OS, OP, RA, and IDD (Figure 1 and Table 1).

## 3. The Links between m6A and Regulated Cell Death

The main types of RCD that exist include apoptosis, mitochondrial permeability transition (MPT)-driven necrosis, necroptosis, ferroptosis, pyroptosis, parthanatos, entotic cell death, NETotic cell death, lysosome-dependent cell death, autophagy-dependent cell death, and immunogenic cell death. They have different cellular and molecular mechanisms of action and can lead to different results, such as the potential for triggering an inflammatory response [52]. Among the RCDs that interact with m6A are apoptosis, necroptosis, ferroptosis, pyroptosis, NETotic cell death, autophagy-dependent cell death, and immunogenic cell death [53]. RCD is an important biological process that plays a key role in the evolution of many diseases, including endocrine disorders, cardiovascular disease, and cancer. Increasing evidence suggests that m6A regulation interacts with almost all pathways involved in RCD. m6A regulation in RCD has attracted attention in the field of disease research, and its impact on RCD undoubtedly provides some new insights into the management of related diseases.

### 3.1. m6A Modification and Apoptosis

**Figure 2:**
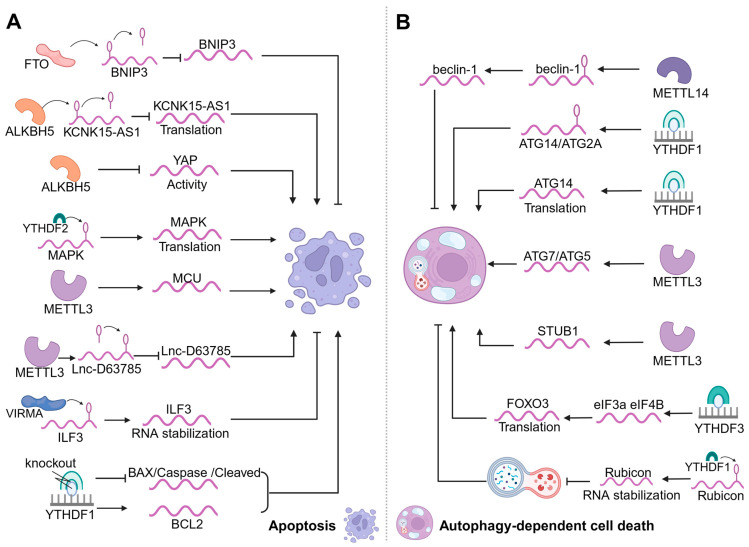
Functions of m6A in apoptosis and autophagy-dependent cell death. (**A**) In apoptosis, writers (ALKBH5/METTL3) and readers (YTHDF1/YTHDF2) act on relevant genes and promote apoptosis by affecting the translation, activity, degradation, and stability of these RNAs. The eraser (FTO) and writer (VIRMA) inhibit apoptosis by regulating the expression and stability of related RNAs. (**B**) In autophagy-dependent cell death, writers (METTL3/METTL14) and readers (YTHDF1/YTHDF3/IGF2BP1) regulate related genes and contribute to autophagy-dependent cell death by influencing the translation and expression of these RNAs. In addition, the reader (YTHDF1) inhibits autophagy-dependent cell death by modulating the stability of related RNAs.

Apoptosis is the predominant form of RCD, which begins with the shrinkage of the cell and the condensation of the nucleus and a chromatin called pyknosis. Next comes the fragmentation of the nucleus and a chromatin called karyorrhexis. Cell membrane blebbing occurs, before the budding of the cell into a series of membrane-bound structures called apoptotic bodies [54]. One study suggested that in the apoptotic pathway, m6A modification may affect apoptosis by regulating the methylation and demethylation of relevant genes, modifying RNA stability, and recognizing transcriptional and translational processes [54]. METTL3 downregulation is involved in colistin-induced nephrotoxicity through methylation modification via Keap1/Nrf2-signaling-pathway-mediated apoptosis [55] and the perinatal knockdown of METTL3 with *Alb-Cre*-induced apoptosis in hepatocytes [56]. METTL3 was also found to be upregulated in human lens epithelial cells (HLECs) from diabetic cataract (DC) patients. The knockdown of METTL3 promotes the proliferation of HLECs and inhibits apoptosis [57]. METTL3 methylates increase mitochondrial calcium single transporter protein (MCU) translation and expression, leading to apoptosis [58]. METTL3 also plays an important role in the regulation of apoptosis by inducing the methylation of Lnc-D63785m6A, decreasing the expression of Lnc-D63785 and leading to neuronal cell death and apoptosis [59]. In human airway epithelial cells, METTL3 regulates apoptosis by targeting the *OSGIN1* gene [60]. In bladder urothelial carcinoma cells, the downregulation of METTL14 resulted in decreased m6A abundance and increased apoptosis [61]. The addition of the methyltransferase METTL3 and METTL14 increased apoptosis and decreased cell viability in SH-SY5Y cells [62]. It was shown that HNRNPA2B1 affects apoptosis by recognizing the m6A site of *ILF3* and enhancing the stability of its transcripts. HNRNPA2B1 gene deletion induces apoptosis in MM cells [63]. In contrast, FTO inhibits apoptosis by demethylating *BNIP3* mRNA and reducing the expression of the pro-apoptotic gene *BNIP3* [64]. ALKBH5 triggers the m6A demethylation of *KCNK15-AS1* and contributes to the upregulation of it. And *KCNK15-AS1* promotes PC cell apoptosis [65]. ALKBH5 also inhibits tumor growth and tumor migration by decreasing the activity and expression of *YAP* in NSCLC, ultimately leading to apoptosis in NSCLC cells [66]. The knockdown of ALKBH5 triggers apoptosis in MM cells and inhibits their growth in vitro [67], and the knockdown of IGF2BP1 resulted in increased apoptosis in Beas-2B cells [68]. Similarly, YTHDF1 knockdown decreased the levels of pro-apoptotic proteins BAX, caspase-3, and cleaved caspase-3 in the tissues of mice and then inhibited apoptosis [69]. One study revealed that YTHDF2 interacts with proteins in the MAPK pathway, selectively promoted apoptosis in TNBC cells in YTHDF2-deficient solid tumors [70]. Taken together, we found that the m6A–apoptosis axis is primarily writer-activated. m6A–apoptosis has already played a regulatory role in many diseases, and its role in MSD deserves further investigation (Figure 2A).

### 3.2. m6A Modification and Autophagy-Dependent Cell Death

Autophagy-dependent cell death is another important form of RCD, involving a series of sequential steps including autophagosome formation, autophagosome maturation, fusion with lysosomes to form autophagosomes, and subsequent degradation. Throughout the autophagy-dependent cell death process, it is tightly regulated by various *autophagy-related genes* (*ATGs*). m6A modification can affect autophagy-dependent cell death by regulating the target molecules involved in autophagy-dependent cell death regulation. In addition, m6A modification can also affect autophagy-dependent cell death through methylation and demethylation, affecting the transcription, translation, and expression of related genes and the stability and degradation of mRNAs. Thus, m6A regulators can inhibit autophagy-dependent cell death by promoting the mRNA degradation of *ATGs* [71]. METTL14 promotes the methylation modification of the m6A of the *beclin-1* transcript and enhances *beclin-1* expression, and this modification promotes the mRNA degradation of transcripts and inhibits autophagy-dependent cell death [72]. The knockdown of METTL14 can have a protective effect on injured infarcted cells by promoting autophagy-dependent cell death, reducing apoptosis and inflammation [73]. METTL3 positively promotes autophagosome production through the upregulation of *ATG5* and *ATG7*, which enhances autophagic processes [74]. METTL3 also regulates autophagy-dependent cell death by stabilizing *STUB1*, which leads to an increase in *STUB1* expression and thus enhances the clearance of autophagic p-Tau [75]. YTHDF1 directly recognizes the m6A methylation of *ATG2A* and *ATG14*, which leads to autophagy-dependent cell death promotion [76]. It also works importantly in autophagy-dependent cell death by affecting the transcription of *ATG14* and promoting the translation of the autophagy-associated gene *ATG14*, which ultimately enhances autophagy-dependent cell death [77]. The YTHDF1 protein’s interactions with METTL3 can promote the stability of m6A-tagged *Rubicon* mRNA. Moreover, *Rubicon* inhibits autophagosome–lysosome fusion, which in turn inhibits autophagy-dependent cell death in nonalcoholic fatty liver disease [78]. YTHDF3 promotes autophagy-dependent cell death by recruiting *eIF3a* and *eIF4B* to assist *FOXO3* translation [79]. In diabetic skin, YTHDC1 regulates autophagy-dependent cell death by modulating the stability of SQSTM1 nuclear mRNA, and endogenous YTHDC1 knockdown inhibits epidermal autophagy-dependent cell death [80]. We found that m6A’s link with autophagy-dependent cell death also acts mainly on writers, affecting erasers and readers less with regard to autophagy-dependent cell death. Therefore, other effects of m6A-mediated cellular autophagy-dependent cell death are still possible but remain to be discovered (Figure 2B).

### 3.3. m6A Modification and Ferroptosis

**Figure 3:**
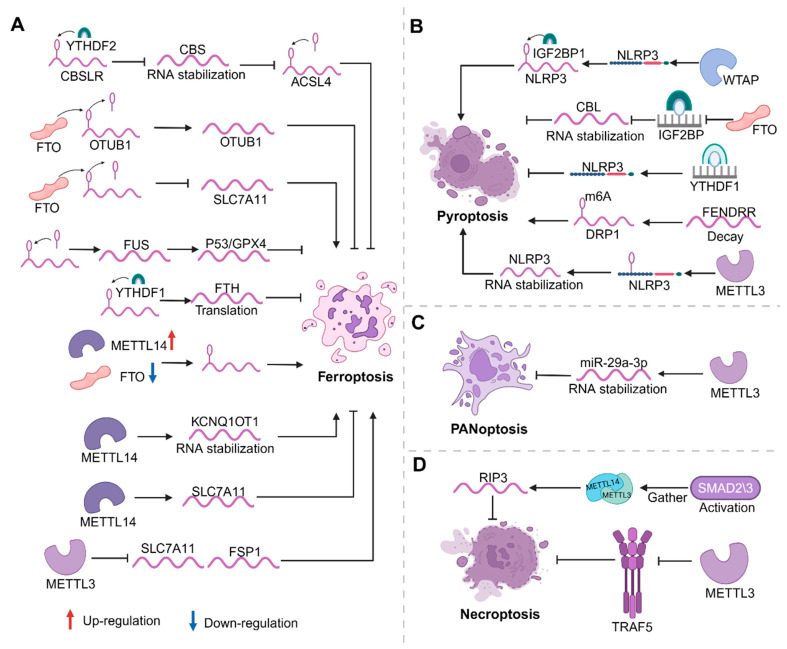
Roles of m6A in ferroptosis (**A**), pyroptosis (**B**), PANoptosis (**C**), and necroptosis (**D**). (**A**) In ferroptosis, writers (METTL3/METTL14) and the eraser (FTO) act on relevant genes and promote ferroptosis by affecting the translation, activity, expression, and stability of these RNAs. The eraser (FTO), writer (METTL14), and readers (YTHDF1/2) inhibit ferroptosis by regulating the expression and translation of related RNAs. (**B**) In pyroptosis, writers (METTL3/WTAP) contribute to pyroptosis by promoting RNA stability and the reader (YTHDF1) and eraser (FTO) can inhibit pyroptosis by suppressing RNA stability. (**C**) In PANoptosis, the writer (METTL3) represses PANoptosis by promoting RNA stability. (**D**) In necroptosis, writers (METTL3 and METTL14) can promote RNA expression, which consequently inhibits necroptosis.

Ferroptosis results from excessive accumulation of lipid peroxides due to the disruption of intracellular metabolic pathways. It is a common form of cell death and is closely related to cellular iron metabolism and lipid homeostasis. Ferroptosis regulated by a lot of cellular metabolic pathways, including iron metabolism, redox homeostasis, mitochondrial activity, and amino acid, lipid, and glucose metabolism, as well as a variety of associated disease signaling pathways. m6A modification regulates ferroptosis in disease by affecting the degradation, stability, shearing, and maturation of mRNAs through methylation and demethylation, as well as by affecting the expression of related genes involved in these processes. It was found that the upregulation of the methylase METTL4 and downregulation of the demethylase FTO improved the overall level of m6A modification that induces ferroptosis. The identification of YTHDF1 as an essential m6A reader protein that stabilizes *BECN1* mRNA suggests that the knockdown of YTHDF1 prevents *BECN1* plasmid-induced HSC ferroptosis [81,82]. METTL3 inhibits the expression of the important ferroptosis regulatory proteins SLC7A11 and FSP1, thereby promoting ferroptosis in human aortic smooth muscle cells (HASMCs) [83]. Meanwhile, METTL14 controls ferroptosis by increasing the stability of *KCNQ1OT1*. This in turn promotes iron uptake and lipid reactive oxygen species production, ultimately initiating ferroptosis [84]. METTL14 also inhibits ferroptosis by targeting the *SLC7A11* mRNA by the induction of lipid peroxidation products and lethal reactive oxygen species (ROS) through iron dependence [85]. A study found that GNRa-CSP12 selectively inhibited the growth of leukemia cells by inhibiting global m6A RNA methylation-regulated ferroptosis [86]. FTO acts as an m6A demethylase to remove m6A modification from *OTUB1* transcripts, promoting *OTUB1* expression and effectively inhibiting ferroptosis [87]. FTO can also promote ferroptosis by downregulating the expression of the ferroptosis suppressor gene *SLC7A11* through m6A demethylation [88]. The increased methylation modification of m6A promotes the expression of *P53* and *GPX4*, upregulates *GSH*, downregulates *ROS*, and inhibits ferroptosis [89]. YTHDF2 interacts with lncRNA *CBSLR* to destabilize *CBS* mRNA and reduce *CBS* expression. Ultimately, this protects gastric cancer cells from ferroptosis in a hypoxic tumor microenvironment [12]. YTHDF1 controls ferroptosis in lung cancer cells by promoting the translation of ferritin (FTH) in an m6A-dependent manner. The deletion of YTHDF1 induces FTH-mediated ferroptosis, whereas the overexpression of FTH counteracts the effects induced by the knockdown of YTHDF1 [90]. *NKAP* acts as a novel ferroptosis inhibitor and the downregulation of *NKAP* directly induces ferroptosis in glioblastoma cells [91]. Ferroptosis is always a hot topic of research today, and m6A interaction with RCD can regulate many genes and pathways. The regulation of m6A-regulated ferroptosis on other related genes and pathways deserves further exploration (Figure 3A).

### 3.4. m6A Modifications and Other Regulated Cell Deaths

In addition to the recognized classical forms of RCD, relationships between m6A modifications and several emerging forms of RCD (pyroptosis, necroptosis, PANoptosis, immunogenic cell death, and NETotic cell death) are gradually being discovered. This reveals new perspectives on how m6A modifications regulate RCD and influence disease outcomes. Pyroptosis is a newly identified and proven form of RCD recognized by its reliance on inflammatory caspases (mainly caspase-1, -4, -5, -11) and the release of high levels of pro-inflammatory factors. m6A modifications can modulate inflammation-related targets such as *caspase-1* and *NLRP3* and thus have an impact on pyroptosis in disease. In addition, m6A modifications regulate pyroptosis through methylation, affecting mRNA stability and influencing gene expression. FTO reduces *CBL* mRNA stability by inhibiting IGF2BP3-mediated m6A methylation, ultimately inhibiting pyroptosis [92]. m6A-induced FENDRR decay promotes the pyroptosis of hypoxic pulmonary artery endothelial cells (HPAECs) by regulating DRP1 promoter methylation [93]. Furthermore, it was also found that human umbilical cord mesenchymal stem cell (hucMSC) exosomes were effective in enhancing myeloid (NP) cell viability and protecting the cells from pyroptosis by acting on METTL14 [94]. And the METTL3 m6A regulator was observed to increase the m6A modification level of *NLRP3* mRNA in trophoblasts and increase the stability of *NLRP3* mRNA. It then promoted trophoblast cell pyroptosis [95]. METTL3 knockdown reduces the m6A modification of *NLRP3* mRNA, which attenuates BPDE-induced trophoblast cell pyroptosis. Proteins produced in response to WTAP with IGF2BP1 contribute to the formation of *NLRP3* inflammatory vesicles, which further regulates *caspase-1*-dependent inflammation and pyroptosis [96]. YTHDF1 promotes *NLRP3* ubiquitination by enhancing *WWP1* expression, which inhibits *caspase-1*-mediated pyroptosis, ultimately leading to the amelioration of sepsis [97]. METTL3 overexpression can attenuate high-glucose-induced RPE cell pyroptosis [98]. The silencing of METTL3 alleviated the inflammatory cytokine spurt induced by Kupffer cell pyroptosis in ASH mice (Figure 3B). In vitro, the silencing of METTL3 with lentivirus reduced pyroptosis by affecting the splicing of pri-miR-34 [99].

Necroptosis is a pro-inflammatory modulated necrosis usually caused by overwhelming intracellular and extracellular insults, such as ROS, TNFα, and FAS ligand production. Its morphological characteristics are comparable to those of non-regulatory necrosis, manifested by membrane rupture, organelle swelling, and cytoplasmic vacuolization, with the release of DAMP (damage-associated molecular pattern) playing a role [100]. In vivo study has shown that interfering with METTL3 and METTL14 attenuates the pathological process of necroptosis, the inflammatory response, and AAA in VSMC [101]. Enhanced METTL3-mediated m6A modification inhibits *TRAF5*-mediated necroptosis, and *TRAF5* absence significantly increases the number of oxygen-induced necroptosis cells (Figure 3D) [102]. PANoptosis is recognized as an inflammatory, lysogenic cell death pathway driven by caspases and RIPKs and is controlled by the PANoptosome complex, setting it apart from other cell death pathways. To date, several PANoptosome complexes (including ZBP1-, AIM2-, RIPK1-, and NLRP12-PANoptosomes) have been identified. In addition, PANoptosis has been associated with infectious and inflammatory diseases, cancer, and disturbances in homeostasis. Thus, targeting its molecular components offers great potential for therapeutic development [103]. It is a unique mode of inflammatory cell death that involves a common combination of cellular pyroptosis, apoptosis, and necroptosis, and it can be facilitated by a multifaceted PANoptosome complex formed through the integration of components derived from other cell death modalities [104]. METTL3-mediated m6A modification enhances *miR-29a-3p* expression and stability, which can effectively inhibit inflammation and PANoptosis in alveolar epithelial cells through interaction with *TNFR1* (Figure 3C). *miR-29a-3p* agomir interacting with METTL3 via injection has been shown to significantly reduce inflammatory factor levels present in lungs, thereby attenuating PANoptosis in alveolar epithelial cells [105]. Immune cell death (ICD) belongs to a special type of apoptosis that enables an immunocompetent host to mount a specific immune response to antigens associated with dead cells [106]. Studies show that it currently serves a role in cancer [100] and MM [107]. In addition, other RCDs with m6A, including NETotic cell death [108] and oxeiptosis [109], now also play a role primarily in cancer. In summary, most of the new studies on RCD and m6A focus on tumors, and there are few studies on other diseases. Whether they are related to the pathophysiological mechanisms of other diseases is worth further exploration.

## 4. m6A and Regulated Cell Death in the Musculoskeletal System

The human body is a complex organism whose total mechanical properties are achieved by an interconnected musculoskeletal network. The musculoskeletal system mainly includes muscle, bone, and cartilage. The cardiac muscle allows the heart to maintain normal autoregulation as well as conduction. Skeletal muscle contraction is essential for the movement of our musculoskeletal system. Smooth muscles allow the movement and deformation of organs by shortening and creating tension. Bone provides support, protection, hematopoiesis, movement, and storage. It also has a vital role in the human body. Cartilage is crucial for load bearing, the lubrication of joints, and force absorption in the body. In muscle, bone, and cartilage, we found that m6A regulators (writers and erasers, as well as readers) mediated the downstream targeting and thus the regulation of RCD. The association between m6A and RCD pathways will provide new ideas for the treatment of musculoskeletal disorders.

### 4.1. m6A and Regulated Cell Death in Muscle

**Figure 4:**
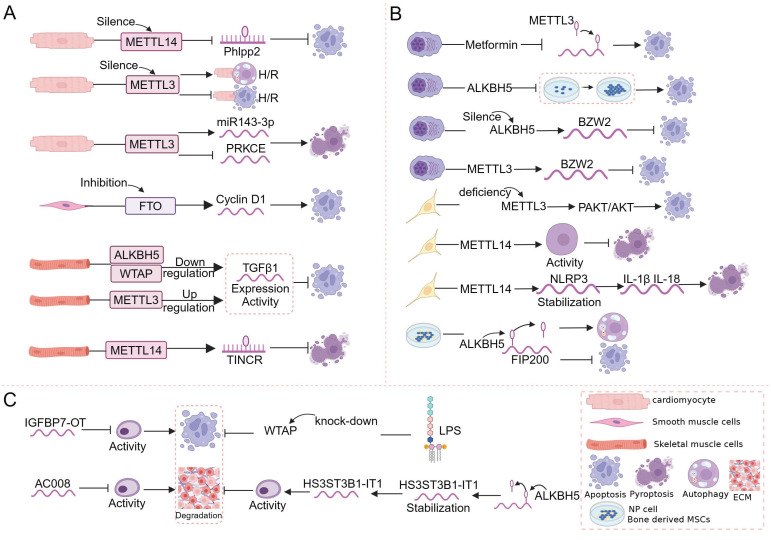
Functions of m6A and RCD links on muscle (**A**), bone (**B**), and cartilage (**C**). (**A**) m6A-related genes act mainly by regulating apoptosis, autophagy-dependent cell death, or pyroptosis in muscle (cardiomyocytes, smooth muscle cells, and skeletal muscle cells). The writers (METTL3/METTL14/ALKBH5/WTAP) primarily assume a role in this process, while the eraser (FTO) also exerts a certain influence. (**B**) They also act by regulating apoptosis or pyroptosis or autophagy-dependent cell death in bone (myeloma cells, OS cells, and mesenchymal stem cells). In this process, it is mainly the writers (METTL3/METTL14/ALKBH5) who play a role. (**C**) In addition, in cartilage, m6A modifications exert their effects mainly by affecting apoptosis through the writers (METTL3/ALKBH5/WTAP) in chondrocytes.

Numerous studies have found that the relationship between m6A and RCD affects the biological processes of a wide range of cells, including cardiomyocytes, smooth muscle cells, and skeletal muscle cells. These changes lead to further alterations in the musculoskeletal system. In cardiomyocytes, m6A mainly participates in the modulation of cardiomyocyte apoptosis, autophagy-dependent cell death, and pyroptosis. METTL14 inhibition has shown remarkable protective action in oxygen glucose deprivation/reperfusion (OGD/R)-induced apoptosis in cardiomyocytes. The silencing of METTL14 inhibited *Phlpp2* mRNA m6A modification, which in turn inhibited apoptosis [110]. Related research also pointed out that METTL3 is a major contributor to m6A abnormalities. In H/R-treated cardiomyocytes, the silencing of METTL3 enhanced the autophagy flux and inhibited apoptosis. Nevertheless, the overexpression of METTL3 or repression of the RNA demethylase ALKBH5 produced the contrary result, demonstrating that METTL3 is a negative modulator of muscle autophagy-dependent cell death [111]. METTL3 affects cardiomyocyte pyroptosis by influencing *miR-143-3p* expression. METTL3 increases *miR-143-3p* expression, and the overexpression of *miR-143-3p* reverses the suppressive impact of METTL3 silencing on pyroptosis in cardiomyocytes [112]. In smooth muscle cells, m6A is mainly engaged in the management of smooth muscle cell apoptosis, and FTO positively regulates the expression of Cyclin D1, which has a significant effect on apoptosis in pulmonary artery smooth muscle cells (PASMCs) [113]. RCD also exerts a major influence in skeletal muscle cells and is an important marker of many muscle-related diseases and pathophysiological processes. In skeletal muscle cells, m6A primarily participates in the control of apoptosis and pyroptosis. Currently, *transforming growth factor β1* (*TGFβ1*) is engaged in numerous cellular processes like differentiation, proliferation, and apoptosis. Related studies found that the knockdown of *TGFβ1* inhibited myoblast proliferation and induced apoptosis. These findings suggest that m6A–RCD is very important in myofibroblast growth and is negatively regulated by m6A–RCD [114]. In addition, the downregulation of ALKBH5 and WTAP favored the inhibition of myofibroblast apoptosis, whereas the inhibition of METTL3 hindered myofibroblast apoptosis. However, the inhibition of METTL14 may have a neutral effect on myoblast apoptosis [115]. In cardiomyopathy (DCM), METTL14 inhibits cellular pyroptosis and DCM progression in an *NLRP3*-dependent manner via the m6A methylation of *TINCR* mRNA [116]. Overall, the m6A–RCD axis plays a crucial role in muscle tissue function (Figure 4A).

### 4.2. m6A and Regulated Cell Death in Bone

The association between m6A and RCD has a crucial effect on bone tissue. Numerous studies have found that the m6A–RCD axis affects the biological functions of various cells such as myeloma (MM) cells, OS cells, and mesenchymal stem cells. These changes lead to further alterations in the musculoskeletal system. In addition to this, RCD (e.g., apoptosis, ferroptosis, autophagy-dependent cell death, necroptosis, and pyroptosis) are cell death processes that play an important function in modifying bone metabolism by defining the fate of osteoblasts. And this process is further affected by the m6A regulation of RCD. It was found that in MM cells and OS cells, m6A is mainly involved in the regulation of apoptosis. Metformin inhibits METTL3-mediated m6A methylation, thereby impeding the proliferation and growth of various myeloma (MM) cells and promoting apoptosis [117]. In vitro and in vivo, METTL3 can also affect the proliferation, apoptosis, and pluripotency of MM cells by speeding up the *YY1* constancy and the development of primary *mir-27a-3p* [118]. METTL3 promotes cell viability and reduces apoptosis through overexpression by positively regulating *BZW2* expression. The *BZW2* pro-apoptotic effect was inhibited by its downregulation, and apoptosis was suppressed by its overexpression [119]. It was found that in NP cells and MSCs, m6A is mainly involved in regulating cellular pyroptosis. The results showed that hucMSC exosomes were effective in increasing NP cell viability and protecting them from pyroptosis by targeting METTL14. METTL14, which is abundant in NP cells from IVDD patients, stabilizes *NLRP3* mRNA. Elevated levels of *NLRP3* led to increased levels of IL-18 and IL-1β and caused pyroptosis and NP cell death [94]. In addition to this, in vitro and in vivo, ALKBH5 inhibition has proven to significantly impede myeloma cell proliferation, reduce invasive, migratory capacity, and promote apoptosis [67,120]. It has also been shown that mRNA modification is reduced and autophagic flux is increased when myeloid cells are co-cultured with bone-derived MSCs. Under co-culture conditions, *FIP200* mRNA demethylation mediated by the RNA demethylase ALKBH5 promoted autophagy flux and attenuated NPC apoptosis under compression [121]. In OS tissues, the upregulation of ALKBH5 decreases m6A mRNA levels in human OS cells, leading to cell apoptosis and cycle arrest [122]. There are also findings suggesting that in knockdown *HNRNPA2B1* MM cells, *HNRNPA2B1* has a critical effect in facilitating MM proliferation and restraining MM apoptosis [123]. Taken together, this research shows that the m6A–RCD axis plays an essential role in bone tissue function (Figure 4B).

### 4.3. m6A and Regulated Cell Death in Cartilage

Much research has found that m6A–RCD links could affect the biological functions of chondrocytes. These changes lead to further alterations in the musculoskeletal system. m6A affects RCD progression by influencing the expression of related factors, which in turn affects chondrocyte. Mechanistic studies revealed that IGFBP7-OT promoted the development of OA by upregulating the expression of IGFBP7. IGFBP7-OT inhibited the viability of cells, promoted apoptosis, and reduced ECM components in human primary chondrocytes. It was also found that IGFBP7-OT and IGFBP7 were upregulated in osteoarthritic cartilage. Flow cytometry analysis showed that the overexpression of IGFBP7-OT significantly promoted apoptosis. The protein level of the anti-apoptotic protein *Bcl-2* was also downregulated by the overexpression of IGFBP7-OT [21]. In other studies, the ALKBH5-mediated demethylation of m6A increased *HS3ST3B1-IT1* RNA stability. It was demonstrated that *HS3ST3B1-IT1* overexpression remarkably enhanced chondrocyte viability, depressed chondrocyte apoptosis, and upregulated extracellular matrix (ECM) proteins, while *HS3ST3B1-IT1* knockdown had the opposite effect [124]. In a chondrocyte injury model of LPS-induced OA, WTAP knockdown enhanced proliferation, prevented apoptosis, reduced extracellular matrix (ECM) degradation, and ameliorated cartilage injury in a mouse model of unstable medial meniscus (DMM)-induced OA. Consistent with this, we discovered that WTAP knockdown significantly decreased caspase-3 activity in LPS-induced chondrocytes. In contrast, WTAP overexpression has the opposite effect [125]. Related studies also demonstrated that FTO-mediated m6A demethylation downregulated *AC008* transcription, whereas lower FTO expression resulted in the upregulation of *AC008* transcription in OA. In addition, *AC008* overexpression reduced chondrocyte viability and induced chondrocyte apoptosis and ECM degradation in vitro, while the knockdown of *AC008* had the reverse effect [20]. Overall, developments regarding the functional role of m6A and RCD on chondrocytes are still relatively few. We think that m6A-mediated RCD has an important role in the function of cartilage tissue. However, more confirmation is needed to demonstrate the effect of the link between m6A and RCD on cartilage function (Figure 4C).

## 5. Links between m6A and Regulated Cell Death in Musculoskeletal Diseases

RCD is thought to be strongly associated with the pathophysiological progression of MSD. RCD can occur in a variety of cells including chondrocytes, synoviocytes, osteocytes, bone marrow stromal cells, hematopoietic stem cells, disc cells, and nucleus pulposus cells. The death of these cells can lead to further deterioration of articular cartilage and joint inflammation in OA, influencing the course and invasion of OS and tumor cell aggregation and disease progression. Recently, it was also found that the link between m6A and RCD further affects the progression of MSD.

### 5.1. Osteoarthritis

**Figure 5:**
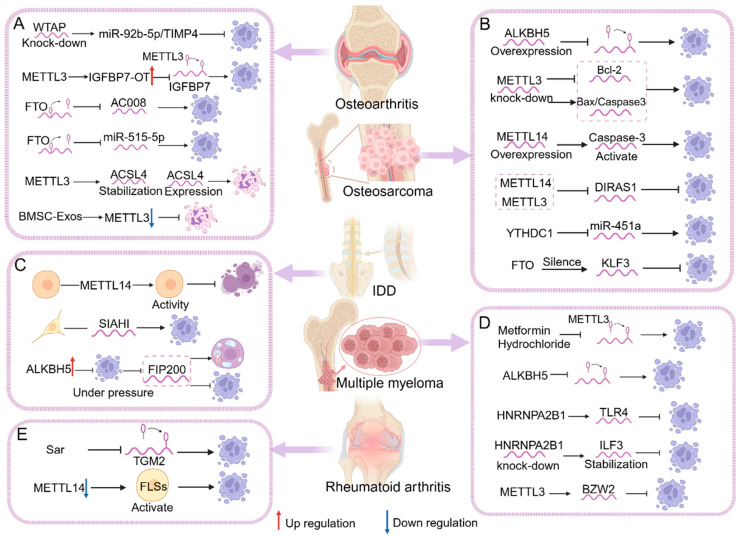
Regulatory role of m6A–RCD in diseases of the musculoskeletal system. (**A**) In OA, m6A (WTAP, METTL3, and FTO) affects OA mainly through the effect of apoptosis or ferroptosis on chondrocyte viability, protein ubiquitination and degradation, and synovial inflammation, which in turn affects disease course and progression. (**B**) In OS, m6A (ALKBH5, METTL3, METTL14, and FTO) affects OS mainly through the effect of apoptosis on chondrocyte cell viability, cell migration and colony formation, anti-tumor activity, and the expression of anti-apoptotic and pro-apoptotic proteins, thereby regulating cell survival and tumor progression. (**C**) In IDD, m6A (METTL14 and ALKBH5) affects intervertebral disc structure mainly through apoptosis, autophagy-dependent cell death, or pyroptosis, altering the progression of degeneration. (**D**) In MM, m6A (METTL3, ALKBH5) affects MM cell proliferation, and cell stemness, invasion, and migration capacity mainly through apoptosis, which in turn regulates MM cell survival. (**E**) In RA, m6A (METTL14) affects the inflammatory response in RA mainly through apoptosis.

OA is a joint disorder recognized by cartilage degeneration and inflammation, leading to impaired joint function and pain. RCD is thought to be strongly associated with the pathophysiological progression of OA. In OA, RCD can occur in various types of cells, including chondrocytes, synoviocytes, and osteocytes. The death of these cells can be triggered by apoptosis or other RCD pathways, leading to further deterioration of articular cartilage and joint inflammation. Significantly elevated levels of m6A modification in articular cartilage and synovial tissues of patients with OA have been found to correlate with the development of RCD. Alterations in m6A modification can affect chondrocyte viability and the process of RCD, which can regulate the cell survival and death signaling pathways that influence disease progression. Tang et al. found that METTL3-mediated m6A modification was closely related to chondrocyte viability and apoptosis. METTL3 could increase RNA stability and enhance IGFBP7 transcription, thereby inhibiting chondrocyte viability and accelerating chondrocyte apoptosis by regulating *Bax*, *caspase-9*, *caspase-3*, *Bal-2*, and *PARP*, ultimately promoting OA progression [21]. And METTL3 suppression dramatically diminished *IL-1b*-induced apoptosis. Therefore, investigation identified a positive effect of METTL3 in chondrocyte apoptosis during inflammation cytokine *IL-1b* stimulation. The METTL3–apoptosis axis promotes the progression of OA [126]. Conversely, the upregulation of METTL3 elevates m6A levels and is followed by enhanced *p62* and *GATA4* expression, whose modification has an essential role in autophagy-dependent cell death. METTL3 links with autophagy-dependent cell death to promote OA progression [127]. In addition, BMSC-Exos limits chondrocyte ferroptosis by downregulating METTL3, which is abrogated by METTL3 overexpression. BMSC-Exos prevents OA progression by disrupting the METTL3–ferroptosis axis [128]. It was also revealed that WTAP was upregulated in OA chondrocytes. The results indicated that the knockdown of WTAP greatly reduced LPS-induced apoptosis in OA chondrocytes. Moreover, they proved that the WTAP–apoptosis axis inhibited the deterioration of OA [125]. YTHDF2 regulates the expression of *CircRERE*, which in turn regulates the apoptosis of HCs. *CircRERE* silencing increased the apoptosis of HCs, whereas its overexpression reversed the IL-1β-induced apoptosis of HCs. Therefore, it eases the progression of OA [129]. FTO affects cell viability and apoptosis by regulating *miR-515-5p* expression. When *miR-515-5p* is inhibited, cell viability decreases and apoptosis is promoted by regulating *Bax*, *cleaved caspase-3*, and *Bcl-2* protein levels. The FTO–apoptosis axis can alleviate OA to some extent [130]. Yang et al. found that FTO can also regulate cell viability and apoptosis by affecting *AC008* expression. Decreased FTO expression resulted in the upregulation of *AC008* transcription in OA and increased the apoptosis rate after *AC008* overexpression. This also upregulates the expression of pro-apoptotic proteins such as *cleaved caspase-3*, *cleaved PARP*, *cleaved caspase-9*, and *Bax*. Finally, the FTO–apoptosis axis accelerated OA progression in vivo [20]. Overall, these studies show evidence of the link between RCD and m6A in the pathophysiological process of OA and provide new directions for further research (Figure 5A).

### 5.2. Osteosarcoma

OS is the third most common malignant tumor in children and adolescents. The most common sites of origin are the distal femur, proximal tibia, and proximal humerus. Patients usually present with pain, swelling, localized enlargement of the limb, and occasionally pathological fractures [131]. m6A serves a crucial role in many biological procedures, and it is one of the most common and abundant mRNA modifications [122]. Increasing data demonstrate that m6A methylation is emerging as a pathway to influence carcinogenesis and tumor progression through the regulation of RCD, and m6A mRNA methylation affects stem and tumor cell growth and proliferation by affecting RCD regulation. Through the action of RCD, m6A can influence the course and invasion of OS via these effects. METTL3 overexpression inhibits cell proliferation and promotes apoptosis. In OS, the knockdown of METTL3 significantly downregulated the expression of the anti-apoptotic protein *Bcl-2*, whereas the expression of the pro-apoptotic proteins cleaved *caspase 3* and *Bax* was markedly upregulated. It has been shown that the METTL3–apoptosis axis inhibits the growth and metastatic ability of OS cells [132]. In OS cells, METTL3 is preferentially upregulated and m6A modification is increased. And the suppression of METTL3 can effectively reduce OS cell apoptosis. This process can promote OS progression [133]. METTL3 can also positively regulate *DRG1* expression and the silencing of *DRG1*, significantly inhibiting cell viability while increasing apoptosis. In summary, the METTL3–apoptosis axis inhibits OS progression [134]. In addition, analysis revealed that METTL14 and METTL3 overexpression reduced *DIRAS1* expression, and the silencing of *DIRAS1* blocked apoptotic ability. *DIRAS1* overexpression increased *Bax* and *cleaved caspase-3* expression and promoted apoptosis, which was significantly reversed by METTL14 and METTL3 overexpression. One study found that the binding of METTL14 and METTL3 with apoptosis can further promote the malignant progression of OS cells [135]. Interestingly, the overexpression of METTL14 in OS cells inhibits cell invasion and migration and promotes apoptosis through the activation of *caspase-3*, which could be a possible strategy for the treatment of OS. Finally, the overexpression of METTL14 facilitated OS cell apoptosis and slowed down tumor progression [136]. Yang et al. found that lowering m6A methylation levels in human OS cells by ALKBH5 overexpression can promote cycle arrest and cell apoptosis and suppress human OS growth in vivo [122]. Results showed that the ALKBH5–apoptosis axis can inhibit the progression of OS [137]. In OS, the increased expression of FTO promoted OS cell invasiveness and inhibited OS apoptosis. Therefore, the FTO–apoptosis axis promotes OS progression [138]. In another study, YTHDC1 3′UTR interacted with *miR-451a* in OS cells. It was found that the upregulation of *miR-451a* increased the rate of apoptosis, while the downregulation of *miR-451a* produced the opposite effect. Collectively, *miR-451a* interacting with YTHDC1 could suppress OS cell migration and proliferation and facilitate OS cell apoptosis [139]. In addition to this, the survival rate of patients with OS has improved in recent years, but the cure rate for OS patients remains low due to rapid course of tumors. Currently, the effect of m6A mRNA methylation as well as its potential mechanism in human OS are still controversial. Therefore, the effect of m6A interaction with RCD on OS deserves further investigation (Figure 5B).

### 5.3. Multiple Myeloma

MM is a malignant plasma cell disease characterized by the clonal proliferation of abnormal plasma cells that accumulate in the bone marrow and form tumors. RCD has a crucial effect on the development of MM. In this case, RCD can occur in plasma cells and other related cell types, including bone marrow stromal cells and hematopoietic stem cells. The death of these cells can be triggered by apoptosis or other forms of RCD pathways. Defects in RCD may lead to the accumulation of tumor cells and disease progression. m6A modifications can regulate RCD in MM through a variety of pathways, and m6A regulates the process of RCD by affecting the expression of its own or other related genes to promote or inhibit the progression of MM disease. And it is mainly the association between writers and apoptosis that has an important role in this process. One study found that the inhibition of METTL3 expression by metformin in turn affects the course of RCD, which ultimately affects MM progression. It promotes apoptosis in a dose-dependent manner. Apoptosis was significantly reduced after METTL3 upregulation, which was reversed by metformin intervention. This process could ameliorate the course of MM disease [117]. Other results showed that METTL3 expression was positively correlated with *BZW2* and was highly expressed in MM bone marrow specimens, which was downregulated to promote apoptosis, while its overexpression inhibited apoptosis in MM cells. In conclusion, METTL3 mediates the m6A methylation of *BZW6* and interacts with apoptosis to promote MM progression [119]. The depletion of METTL3 leads to the strong repression of cell growth and accelerated apoptosis. Similarly, METTL3 overexpression could promote cell viability and diminish apoptosis. Thus, the results validate the oncogenic role of METTL3 interacting with apoptosis in MM [118]. Observations of knockdown *HNRNPA2B1* MM cells suggest that *HNRNPA2B1* plays a key role in inhibiting MM cell apoptosis. The data suggest that *HNRNPA2B1* facilitates tumor procedure in MM by interacting with apoptosis [123]. *HNRNPA2B1* depletion inhibited cell proliferation and induced apoptosis in MM cells. *HNRNPA2B1* increases the stability of *ILF3*, one of the most important targets of *HNRNPA2B1* in MM, and *ILF3* inhibits apoptosis in MM cells. The knockdown of *HNRNPA2B1* reversed its inhibitory effect and became apoptosis-promoting. Therefore, the *HNRNPA2B1*–apoptosis axis promoted MM progression [63]. The knockdown of ALKBH5 caused apoptosis and repressed the growth of MM cells in vitro. In conclusion, these data proved that the ALKBH5–apoptosis axis has a vital effect on MM tumorigenesis and inhibits tumor development and progression [67]. In both MM patients and MM cell lines, another study found that ALKBH5 was highly expressed. ALKBH5 deficiency increased the rate of apoptosis. Mice injected with ALKBH5 cells showed a significant decrease in *pAKT*, *Ki67*, and *BCL-2* and an increase in *caspase-3* and *BAX* activity [120]. There is currently no cure for MM. Although improvements have been made with recently discovered antimyeloma drugs, such as daratumumab and elotuzumab, most patients still relapse [107]. The mechanism of MM pathogenesis is very comprehensive, and it is of great biological, medical, and clinical importance to understand the intrinsic mechanisms associated with the pathogenesis of MM (Figure 5D).

### 5.4. Other Types of Musculoskeletal Disorders

In addition to the diseases mentioned above, RA, IDD, and OP are also common musculoskeletal system disorders. Among them, RA is a chronic autoimmune disease characterized by joint inflammation and destruction. In RA, RCD can occur in a variety of cell types, including synoviocytes, chondrocytes, and osteocytes. The death of these cells can be triggered by apoptosis or other forms of RCD. Currently, the effects of RCD in RA are predominantly apoptotic. RCD exacerbates inflammation and joint tissue damage. m6A modification alterations may be involved in RCD in RA through multiple mechanisms. In addition to this, IDD is a common spinal disorder characterized by structural and functional damage to the intervertebral disc. RCD exerts a crucial effect in the occurrence of IDD. In this condition, RCD can occur in various types of cells within the intervertebral disc, including disc cells, chondrocytes, and nucleus pulposus cells. The death of these cells can be triggered by apoptosis or other forms of the RCD pathway. RCD contributes to the destruction of disc structure and promotes the progression of degeneration. In addition, it was found that m6A interaction with RCD further affects IDD progression. Furthermore, OP is a common bone disorder characterized by reduced bone density and decreased bone mass, leading to weak and brittle bones. In OP, RCD can occur in a variety of cell types, including osteoclasts, bone marrow stromal cells, and osteoblasts. The death of these cells can be triggered through the RCD pathway. Abnormal RCD disrupts the bone remodeling process and accelerates the progression of OP. It was found that m6A interaction with RCD is also critical for the process of OP. In summary, in the onset and progression of RA, IDD, and OP, the disease processes also appear to be inextricably linked to m6A-mediated RCD.

In RA, sarsasapogenin (Sar) has the potential to reduce the m6A methylation of *TGM2* mRNA, thus contributing to the positive effects of Sar in RA-FLS cell cycle arrest and apoptosis, thus ameliorating RA progression [140]. METTL14 expression was strongly evoked in RA synovial tissues (Figure 5E). The knockdown of METTL14 remarkably enhanced apoptosis and suppressed cell migration and invasion. Results suggested that the METTL14–apoptosis axis promotes FLS activity and related inflammatory responses, and the researchers recognized it as a possible idea for treating RA [141]. In IDD, METTL14 can affect cell viability and cellular pyroptosis (Figure 5C). It was found that HucMSC exosomes could effectively improve the survival of NP cells and prevent them from pyroptosis by targeting METTL14 [94]. One study reported that BMSCs under the action of m6A can enhance autophagy-dependent cell death and diminish NPC apoptosis, and this process is reliant on ALKBH5 modification. The knockdown of ALKBH5 resulted in the upregulation of the apoptosis-related proteins, including *cleaved caspase-3*, and the BAX:BCL2 ratio and the downregulation of the autophagy-related proteins *LC3*, *Beclin1*, and *Atg7*. This suggests that ALKBH5 is a novel approach to modulate autophagy-dependent cell death and apoptosis [121]. SIAH1 regulated by METTL3 knockdown reversed apoptosis and elevated levels of inflammation in the NPCs of IDD patients. Finally, the METTL3–apoptosis axis can produce harmful effects in IDD [142]. As METTL16 expression increased in the cells, apoptosis also increased significantly. Results suggested that m6A modification by METTL16 should have an essential role in the mechanism of increased apoptosis and the process of IDD [143]. m6A methylation affects apoptosis in BMSC, osteoclasts, and osteoblasts by modulating the expression of relevant genes. Finally, this in turn affects the onset and progression of OP [16]. A related study found that METTL14-mediated m6A modification post-transcriptionally stabilizes *GPX4* mRNA. The protein *GPX4* is closely related to the concept of ferroptosis, a finding that strongly suggests that the regulation of ferroptosis via m6A may have an impact on the progression of OP [144]. In summary, m6A-mediated RCD has a large impact on the development and progression of numerous musculoskeletal system diseases. However, at present, there are still fewer studies on RA, IDD, and OP. However, the prevalence of all three has not been low. Therefore, the effects of m6A-mediated RCD on RA, IDD, and OP deserve further study.

## 6. Clinical Applications of m6A-Mediated Regulated Cell Death in Musculoskeletal Diseases

In view of the multiple functions of m6A-regulated proteins of RCD, the m6A–RCD axis has emerged as an important mechanism in cancer pathology. The link between the two during the pathologic process will contribute to cancer diagnosis, treatment, and prognosis assessment and will influence the resistance action of drugs. The mechanisms also have clinical applications, focusing on cancer, cardiovascular diseases, and immune disorders. Numerous studies have established that there are many small molecule compounds, and compounds from small molecule plant extracts act by regulating m6A–RCD, which have great therapeutic potential in the treatment of many diseases and are likely to be an important direction for future drug research. Therefore, the study of m6A-mediated RCD can be applied to the diagnosis and treatment of diseases. It was found that in breast cancer cells, the METTL14–apoptosis axis plays an important role in the early diagnosis, treatment, and prognostic assessment of breast cancer [10]. Findings found that METTL3 and YTHDF2 knockdown significantly caused apoptosis, a finding that may be helpful in expanding the potential diagnostic or therapeutic markers for prostate cancer [4]. In addition to this, m6A may be recognized as a potential determinant of the response to therapeutic treatment resistance in several ways, with the control of downstream adaptive responses (autophagy-dependent cell death, apoptosis, etc.) being one of them. In addition to this, numerous studies have found that the link between m6A and RCD has an impact on orthopedic diseases. m6A-mediated RCD may offer an innovative and potentially effective therapeutic target for the treatment of MSD.

Numerous experimental and clinical investigations have affirmed that ATRA and its derivatives are useful and promising agents for the treatment of acute promyelocytic leukemia (APL) and many other kinds of tumor. Relevant ATRA resistance studies have shown that the overexpression of METTL14 in OS cells inhibits cell invasion, proliferation, and migration and promotes apoptosis through the activation of *caspase-3*. METTL14 upregulates MN1 to cause ATRA resistance in OS [145]. It was shown that *ZBTB7C*, the oncogenic protein, may be a key m6A target mediating oncogenic effects. In the OS xenograft model, the STM2457 or siRNA-mediated knockdown of METTL3 significantly reduced *ZBTB7C* abundance. When *ZBTB7C* was overexpressed by lentivirus, the anti-OS effect of STM2457 was reduced. Collectively, the results suggest that METTL3 aberrations and the m6A modification of ZBTB7CD has created an influential epigenetic control loop, which contributes to OS progression, and aiming at the METTL3-*ZBTB7C* axis may offer new insights into promising therapeutic strategies for OS [133]. *HNRNPA2B1* mediates TLR4 to promote MM proliferation and inhibit its apoptosis through m6A modification. Furthermore, *HNRNPA2B1* expression could be remarkably elevated in MM patients and related to a poor prognosis for MM patients [123]. A related study found that the expression of *IGFBP7-OT* and its parent gene, *IGFBP7*, was upregulated and positively associated with osteoarthritic cartilage. *IGFBP7-OT* overexpression highly inhibited chondrocyte viability and promoted chondrocyte apoptosis, whereas the knockdown of *IGFBP7-OT* had the opposite effect. METTL3-mediated m6A modification in OA controls the upregulation of *IGFBP7-OT*. Further mechanistic studies suggest that *IGFBP7-OT* modification contributes to OA progress by mediating the *DNMT1*/*DNMT3a*-*IGFBP7* axis and provides a prospective therapy destination for OA treatment [21]. In osteoarthritic cartilage, it was indicated that ALKBH5 downregulation accelerated *HS3ST3B1-IT1* decay in a YTHDF2-dependent manner. In addition, lowering HS3ST3B1-IT1 caused ubiquitination and promoted *HS3ST3B1* degradation, which ultimately inhibited chondrocyte viability, promoted chondrocyte apoptosis, and reduced ECM components [124]. Therefore, we can deduce that m6A-mediated RCD has a significant impact on musculoskeletal diseases. The m6A–RCD axis might be a possible therapeutic target for the treatment of musculoskeletal disorders. Although there have been numerous studies showing that m6A-mediated RCD serves an essential role in musculoskeletal disorders, the clinical applications are still relatively few, and the current studies are still dominated by research at the molecular level. Therefore, the relationship between m6A and RCD in musculoskeletal disorders deserves further investigation.

## 7. Conclusions and Perspectives

Studies have already shown that the m6A–RCD axis works in cancer prevention, diagnosis, management, prognosis, therapy, and drug resistance and in anti-cancer activity. The m6A–RCD axis has broad prospects in cancer. In MSD, studies have shown that there are already drugs (e.g., STAT, etc.) and polymer compounds that have been used in the clinic. Regulating RCD via m6A modification has been able to improve the occurrence and development of MSD. However, m6A has many regulators, and RCD also contains many different species. Therefore, the link between the two is very complex, and the m6A–RCD axis is a huge regulatory network. To date, researchers have conducted more studies on writers with apoptosis, autophagy-dependent cell death, and ferroptosis, but fewer studies have been conducted on erasers, readers, and other RCDs. In conclusion, the m6A–RCD axis may have therapeutic effects on other diseases besides cancer and musculoskeletal disorders. This axis should be studied in all its dimensions.

## Figures and Tables

**Table 1:** Characteristics and functions of m6A enzymes in m6A modifications.

Category	Enzyme	Characteristics and Functions	Reference
Writers	METTL3	Catalytic subunit, acts as the catalytic core	[27]
	METTL14	Catalytic subunit, acts as an RNA-binding platform and promotes RNA binding	[29]
	WTAP	Regulatory subunit, regulates METTL3/METTL14 complex locates and binds mRNA	[30]
	METTL16	Regulatory subunit, regulates intracellular S-adenosyl methionine levels by dynamically regulating the m6A modification of the small nuclear RNA, *U6*, and target mRNAs	[51]
	VIRMA	Regulatory subunit, recruits the catalytic core component METTL3/METTL14/WTAP to guide regioselective methylation	[31]
	RBM15/RBM15B	Regulatory subunit, consistently methylates adenosine in adjacent m6A residues by binding to the m6A–methylation complex and recruiting it to RNA molecules	[28]
	ZC3H13	Regulatory subunit, anchors WTAP, a virilized protein, and Hakai to the nucleus to promote m6A methylation	[28]
Erasers	FTO	Catalyzes the demethylation of m6A modifications on the mRNA	[33,34]
	ALKBH5	Catalyzes the demethylation of m6A modifications on the mRNA	[33,34]
Readers	YTHDC1	Regulates the mode of alternative splicing; promotes the nuclear exit of m6A-modified mRNA	[37,38]
	YTHDC2	Enhances the translation efficiency or decreases the abundance of its substrate	[37,38]
	YTHDF1	Promotes the translation efficiency of m6A-modified RNA substrates	[37,38]
	YTHDF2	Accelerates the degradation of the m6A-modified transcripts; regulates mRNA stability	[37,38]
	YTHDF3	Cooperates with YTHDF1 to regulate mRNA translation efficiency; mediates mRNA degradation	[37,38]
	HNRNPC	Mediates the alternative splicing of m6A-modified transcripts	[40,41]
	IGF2BP1/2/3	Cooperates with *eIF3* to stabilize target genes and initiate the translation process	[39]
	*eIF3*	Cooperates with IGF2BP1/2/3 to stabilize target genes and initiate the translation process	[37,38]
